# Point-of-Care Assays Could Be Useful for Therapeutic Drug Monitoring of IBD Patients in a Proactive Strategy with Adalimumab

**DOI:** 10.3390/jcm9092739

**Published:** 2020-08-25

**Authors:** Mohamad Cherry, Dominique Dutzer, Yara Nasser, Anne-Emmanuelle Berger, Xavier Roblin, Stephane Paul

**Affiliations:** 1 Department of Immunology, CIC1408, GIMAP EA3064, University Hospital of Saint Etienne, 42055 Saint Etienne, France; Mohamad.Cherry@chu-st-etienne.fr (M.C.); dom_dutzer@hotmail.fr (D.D.); yara.nasser1991@gmail.com (Y.N.); a.emmanuelle.berger@chu-st-etienne.fr (A.-E.B.); 2 Department of Gastroenterology, University Hospital of Saint Etienne, 42055 Saint Etienne, France; xavier.roblin@chu-st-etienne.fr

**Keywords:** POC (Point of Care), therapeutic drug monitoring (TDM), adalimumab, residual trough levels, IBD

## Abstract

The objective of the study was to evaluate whether Point-of-Care (POC) assays are equivalent to ELISAs for measuring residual trough levels of adalimumab (ADA) in a cohort of Inflammatory Bowel Disease (IBD) patients. ADA trough levels obtained by POC assays were used to optimize patients in daily clinical practice. Different assays (three ELISAs (Enzyme-Linked ImmunoSorbent Assay) from two different suppliers and two POC assays) were compared to measure ADA trough levels in a first cohort of 31 IBD patients. All assays revealed a high correlation within the assays, ranging from 0.86 to 0.99. Cut-off values were always higher with ELISAs than with POC assays. Then, a small prospective clinical study with a second cohort of 37 IBD patients was performed to compare POC assays and ELISAs for their ability to optimize patients on the basis of the measured ADA trough levels. The use of a POC assay to monitor ADA trough levels did not improve the follow-up of patients with loss of response, as they were always optimized whatever their ADA residual rate. For patients in clinical remission, a POC assay can be useful in some clinical situations to maintain or de-escalate ADA doses according to the measured trough levels. In conclusion, different assays for ADA monitoring are quite equivalent. A POC assay could be only useful for a proactive strategy for asymptomatic patients with a sub-therapeutic dose of ADA, but new therapeutic thresholds need to be identified.

## 1. Introduction

Adalimumab (ADA), is a fully human anti-TNF (Tumor Necrosis Factor α) monoclonal antibody [1] used for the treatment of rheumatoid arthritis, psoriatic arthritis, ankylosing spondylitis, Crohn’s disease (CD), ulcerative colitis (UC), chronic psoriasis, hidradenitis suppurativa, juvenile idiopathic arthritis, and uveitis [2]. The use of ADA reduces the signs and symptoms of moderate to severe CD [3] and has been approved in the UK since 2009 [4]. It has been also approved by the FDA for the treatment of moderate to severe UC cases in adults [5]. Loss of response to anti-TNFα antibodies such as infliximab (IFX) or ADA has been widely attributed to the induction of specific immunogenicity and to the development of anti-drug antibodies [6]. In this case, dose escalation should be considered if the decrease of the drug concentration is not attributed to immunogenicity failure. In the presence of anti-drug antibodies, a switch to other anti-TNFα agents or to other biotherapies could be beneficial for the patients.

High ADA trough levels correlated with clinical response and mucosal healing, while the presence of anti-ADA antibodies were positively associated with disease activity [7,8,9]. Cut-off values of ADA trough levels have been defined to predict the therapeutic response. The most accepted lower limit is 4.9 μg/mL for the loss of clinical response [10,11]. Regarding the upper limit, a plateau effect in the relationship between ADA serum levels and mucosal healing was observed above 12 μg/mL [12]. Most of the studies and cut-off values used ELISA immunoassays assays. Several methods have been described to measure the level of ADA, including different types of ELISA, radioimmunoassays, homogeneous mobility shift assay (HMSA) based on liquid chromatography (LC), reporter gene assay, and LC coupled with mass spectrometry (MS) [13]. ELISA assays remain the most used to determine both ADA trough levels and anti-ADA antibodies [14]. Two years ago, a new generation of assays for Therapeutic Drug Monitoring (TDM) were first described for IFX. Point-of-Care (POC) assays are based on lateral flow immunochromatography and are now commercially available for IFX and ADA. In principle, they could overcome ELISA limitations such as the duration of the test, the need to work in series of samples, whereas early dosages of anti-TNF therapies yield valuable information regarding endpoint clinical responses [15,16]. Only few studies have compared POC assays and ELISA for IFX determination [17,18], but to date rapid assays for the monitoring of ADA have been scarcely evaluated.

Here, two POC assays (Quantum Blue from Buhlmann (Schönenbuch, Switzerland) and Ridaquick from R-Biopharm (Darmstadt, Germany)) and three ELISA assays (Theradiag (monoclonal vs. polyclonal detection antibody; Croissy Beaubourg, France) and R-biopharm) for monitoring ADA trough levels in Inflammatory Bowel Disease (IBD) patients were compared in a fist cohort of 31 IBD patients. Then, in a prospective second cohort of 37 IBD patients, the clinical interest of a POC assay for monitoring ADA trough levels was evaluated to determine its ability to improve the follow-up of IBD patients.

## 2. Material and Methods

Patients. Residual trough levels of ADA were measured in the serum of a first cohort of 31 IBD patients (Table 1) followed by the gastroenterology department. It was a monocenter (University hospitals of Saint-Etienne), retrospective trial which was approved by the Ethics Committee Board of Saint-Etienne Hospital and the Centre National Informatique et Liberté (CNIL) (Number: 1849323). IBD patients treated with golimumab or IFX were also included as controls. Non IBD patients with high levels of IgM rheumatoid factors were also included as controls, as this has been reported to modify the specificity of this type of assays.

A second prospective clinical study was also performed in a cohort of 37 IBD patients (26 CD and 11 UC) treated with ADA (Table 2). Clinical failure was defined as Harvey–Bradshaw index (HBI) ≥5 associated with fecal calprotectin levels >250 µg/g stools for CD and as Mayo score >5 with an endoscopic sub-score >1 for UC. The clinical status of IBD patients was monitored during 6 months following the decision based or not on the POC assay (Table 2).

Measure of ADA trough levels. Three commercially available ELISA assays from two suppliers (Theradiag (monoclonal vs. polyclonal detection antibody) and R-Biopharm) and two point-of-care methods supplied by R-Biopharm (RIDA^®^QUICK ADA Monitoring) and Buhlmann (Quantum Blue^®^) (Table 3) were compared. The measure of residual trough levels was only performed at the time of the visit for clinical failure or during the classical follow-up visits. All measurements were performed just before the injection of ADA.

### Statistical Analysis

Quantitative analyses were performed using median and interquartile ranges (IQR). A D’Agostino and Pearson omnibus normality test was used to assess the normality of continuous variables. Quantitative differences between ADA trough levels were analyzed using a Wilcoxon test, given the non-normal distribution of the assays. Spearman rank test was used for correlation analysis. ADA trough levels below or above the limit of quantification were considered to be equal to that lower or higher limit. Comparison of all pairs of ADA concentration means, distributions by quartile, was analyzed with one-way analysis of variance and Tukey’s multiple comparison method. Quartile comparison was performed using a one-side Cochrane-Armitage trend. The comparison of the number of fold increases in the threshold of positivity was also realized. A *p* value less than 0.05 was considered statistically significant. GraphPad Prism 5.0 (GraphPad Software, San Diego, CA, USA) was used for all statistical analyses.

## 3. Results

### 3.1. Rapid Assays And ELISAS Are Equivalent To Determine ADA Trough Levels

**Figure 1:**
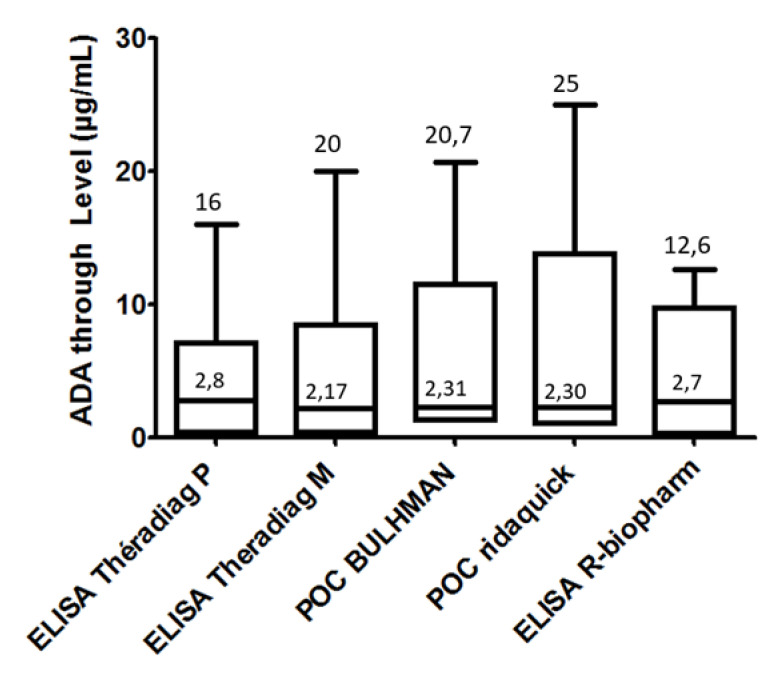
Measure of ADA trough level distribution determined with the different assays. The lines in the boxes represent the median value (µg/mL). POC-Point of Care; ADA: adalimumab.

**Figure 2:**
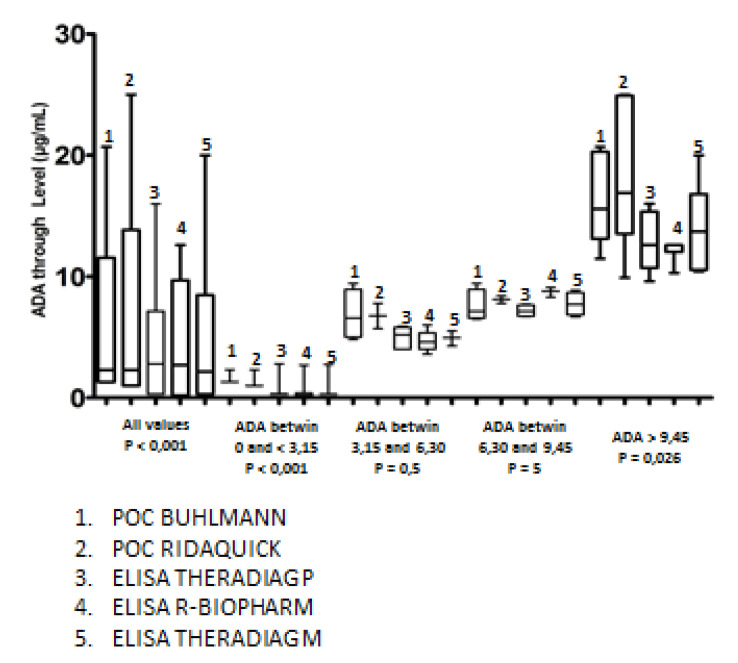
Comparison of interquartile ranges (μg/mL) between the different assays. Four quartiles were defined (0–C3.15, 3.15–6.3, 6.3–9.45, and ≥9.45 μg/mL). These results are without having defined a common upper and lower detection limit for all assays.

**Figure 3:**
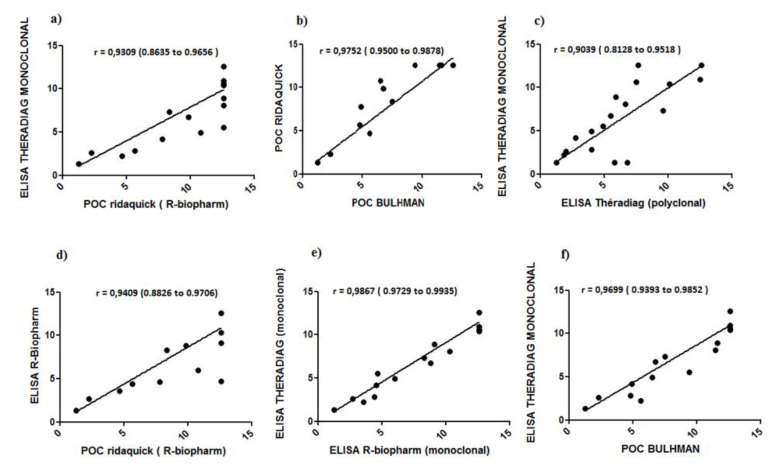
Nonparametric Spearman correlation between the different assays. (**a**) ELISA Theradiag (monoclonal) vs. POC R-Biopharm (Ridaquick), (**b**) POC R-Biopharm (Ridaquick) vs. POC Buhlmann (Quantum Blue), (**c**) ELISA Theradiag (monoclonal) vs. ELISA Theradiag (polyclonal), (**d**) ELISA R-Biopharm (Ridascreen) vs. POC R-Biopharm (Ridaquick), (**e**) ELISA Theradiag (monoclonal) vs. ELISA R-Biopharm (Ridascreen), (**f**) ELISA Theradiag (monoclonal) vs. POC Buhlmann (Quantum Blue).

ADA trough levels were first measured in a first cohort of 31 IBD patients (Table 1). Twenty patients were in clinical remission (20 CD, mean age: 30.5 years, sex ratio M/F: 1.2); 12 patients were had been previously treated with another anti-TNF drug (9 with infliximab); 22 patients were optimized with ADA (40 mg ew). No patients were treated with a combination of immunossuppressive drugs. Medians of ADA trough levels determined by the different assays were compared. Median values were very similar independently of the assay used, ranging from 2.17 to 2.8 μg/mL (Figure 1 and Table 4). To compare the different assays with respect to the concentrations, ADA trough level values were divided into four quartile ranges (0.2–3.15, 3.15–6.3, 6.3–9.45, and ≥9.45 μg/mL). A homogeneous distribution of ADA trough levels was observed in the second and third quartiles (*p* value > 0.05); *p* values were less than 0.05 (*p* < 0.001 and *p* = 0.026, respectively) for the first and fourth quartiles. The distribution of ADA levels was more heterogeneous in the last quartile due to the different ability of rapid assays to quantify high concentrations within a broader linear range for trough determination up to 25 and 35 μg/mL (Figure 1 and Figure 2). A nonparametric correlation with a Spearman *r* coefficient to compare the different results was used, which revealed an agreement ranging from 0.89 to 0.99 between the different ELISAs. The highest *r* coefficient was observed between Theradiag (monoclonal) and R-Biopharm ELISAs. The lowest *r* was observed between Theradiag (polyclonal) and Theradiag (monoclonal) and R-Biopharm ELISAs (0.89). All the other comparisons provided values ranging from 0.86 to 0.99. The results obtained with the two POC assays were perfectly correlated, with an *r* coefficient of 0.99 (Table 4, Figure 3).

### 3.2. A Rapid POC Assay Is Not Useful in All Clinical Situations During the Follow-Up of Adalimumab-Treated IBD Patients

The median ADA through concentrations were compared according to clinical activity pooling the two cohorts of patients and using an ELISA (Theradiag). The median through levels were significantly higher in patients in clinical remission than in those with an active disease (6.9 (3.9–C11) µg/mL versus 1.8 (0–C4.2) µg/mL; *p* = 0.02). Thirty-eight patients were on clinical remission, and 25 presented ADA levels above 4.9 µg/mL; 23 patients were presented clinical activity of their disease, and only 5 presented ADA levels above 4.0 µg/mL. Fourteen patients with a Loss Of Response LOR presented a pharmacokinetic failure defined as ADA levels below 4.9 µg/mL without Anti-Adalimumab Antibody AAA (median: 2.8 µg/mL +/−1.9 µg/mL). Four patients were on immunogenic failure, defined as undetectable ADA levels with high AAA levels (>20ng/mL).

**Figure 4:**
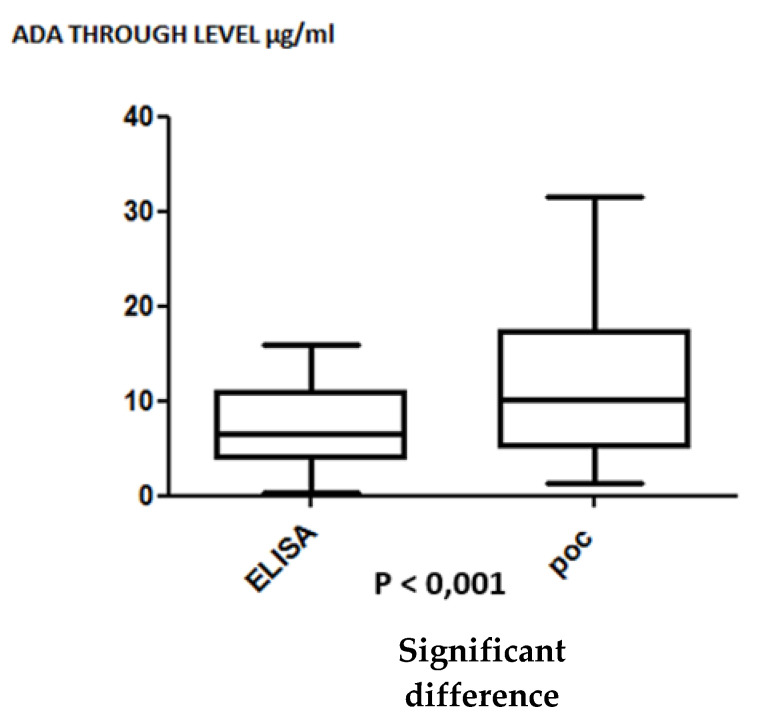
Comparison of adalimumab trough levels in the prospective IBD cohort between ELISA (Theradiag) and POC assay (Quantum Blue; Bulhman).

In the prospective study, ADA trough levels were all quantified using a POC assay and Theradiag ELISA. ADA trough levels were higher than 4.9 µg/mL in 15 patients when using the POC assay and in 14 patients when using ELISA, which is not statistically different. All these patients were optimized regardless of ADA levels. Nine IBD patients responded to therapeutic optimization (5/5 patients and 4/5 patients had an infra-therapeutic dosage with the ELISA and with the POC assay, respectively). For patients with therapeutic rates of ADA, the clinical response rates were equivalent (40% with the POC assay, and 35% with the ELISA). Eighteen patients were in clinical remission, and 2 patients were de-escalated in dose without therapeutic failure in their follow-up. ADA trough levels were in the supra-therapeutic range for those patients (Table 2, Figure 4). In this prospective study, ADA trough levels measured by a POC assay were significantly higher than those obtained by ELISA (Figure 4).

## 4. Discussion

Here, we showed that ELISA and POC assays for monitoring ADA trough levels are globally equivalent. However, it is essential to determine new cut-off values for POC assays associated with the clinical situation of IBD patients. In this study, two rapid POC assays and three commonly used ELISAs were compared for the first time. The usefulness of POC assays and ELISAs was determined in the follow-up of IBD patients treated with ADA. ELISAs are highly comparable but with some discrepancy with high ADA concentrations. Considering our results, the follow-up of a patient needs to be performed using an assay standardized according to WHO standards. The use of external quality controls, now widely available, seems to be crucial for the validation of an assay.

POC assays are similar and relatively well comparable to ELISA assays. However, values of ADA trough levels obtained with POC assays were systematically higher than those determined by ELISAs. This is clearly an issue when applying cut-off values described for loss of response or de-escalation. The measure of repeatability and reproducibility with inter- and intra-assay coefficients of variability showed that SD for ELISA ranged around 5–9%, while SD for POC assays was always around 16–29% [18]. This could be an important issue during the follow-up of patients. For these reasons, ELISAs seem to be more adequate for the follow-up of patients, the trade-off being the time delay in result availability as compared to the rapid POC assay. The use of POC assay could be of interest to clinicians working with whole blood, to obtain results in few minutes without the need of a laboratory and to be able to optimize directly the treatment of patients. This is currently not the case for the tests evaluated here which work only on serum. Quantification limits can be modified by applying a dilution factor to the serum when using the supplier’s first recommended dilution. POC assays’ quantification limits range from 1.3 to 35 μg/mL for Buhlmann and from 1 to 25 μg/mL for R-Biopharm. Therapeutic ADA concentrations have been defined between 4.9 to 12 μg/mL for clinical response and mucosal healing. The determination of high concentrations (higher than 20 µg/mL) could be useful for de-escalation. The delay in the achievement of a result with ELISA is around 100–150 min, but it is necessary to work in series to reduce the cost of the technic. Depending of the lab, it could be an issue to obtain results in a short time interval. The rapid POC assay could deliver results in a shorter time (around 30 min, including serum generation from whole blood). A potential issue also in the use of POCs, particularly in Europe, is the need to accredit them, which is very complicated in the field of delocalized biology.

Our prospective clinical study evaluated for the first time the usefulness of POC assays during the follow-up of ADA-treated IBD patients. In our study, all patients were in loss of response due to an immunogenic failure. This is clearly a limitation of this work, and we cannot conclude about the use of POC assays in this clinical situation. We also assessed the benefits of optimization because it allows more than 30% of positive outcome in the case of symptomatic patients with therapeutic doses. In the case of asymptomatic patients with infra-therapeutic doses, these patients were not optimized according to the TAXIT [19] and TAILORIX [20] studies. In these two studies, ^a^ proactive strategy does not work, and this explains why we did not optimize asymptomatic patients with sub-therapeutic doses unless there was an immunogenic process requiring a combotherapy with an immunosuppressor. However, currently, our strategy has changed following the new PAILOT study [21] and that by Dreesen E et al. [22]: symptomatic patients with infra-therapeutic doses need now to be optimized, as these two randomized studies have proven the effectiveness of a proactive strategy.

**Figure 5:**
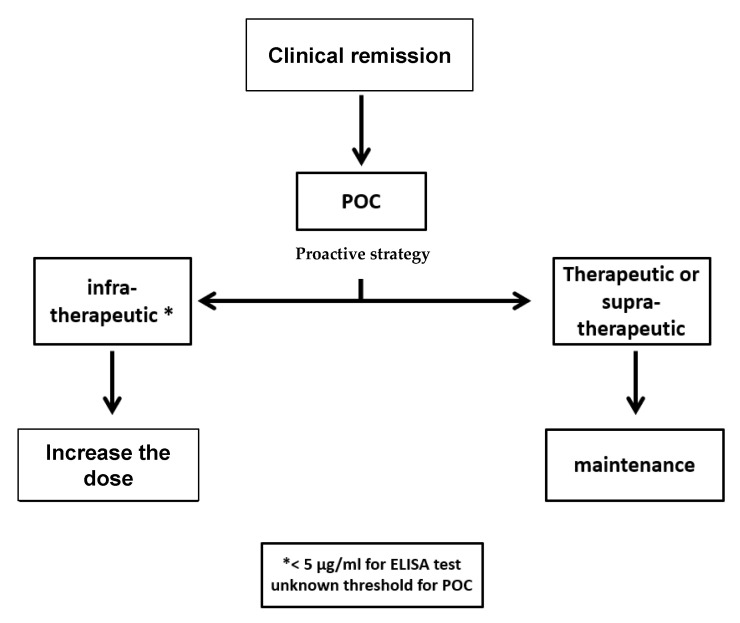
Algorithm for the monitoring of IBD patients in clinical remission.

**Figure 6:**
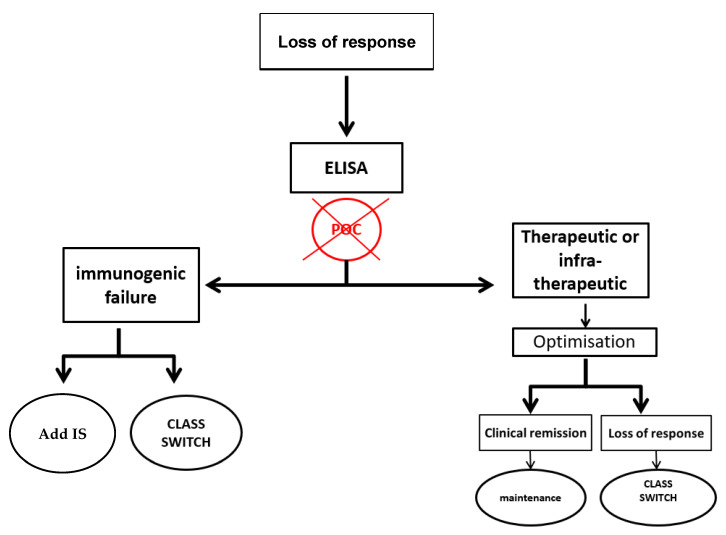
Algorithm for monitoring IBD patients with loss of response.

On the basis of our results, the use of POC assays will probably not change the follow-up of IBD patients. POC assays as ELISAs indicate if a patient has a therapeutic or infra-therapeutic dose, but whatever the result, the patient will be optimized. Moreover, the follow-up of patients requires a period of few weeks to see their clinical evolution after changing the dose regimen. We would like to propose two therapeutic algorithms based on TDM with POC assays or ELISAs (Figure 5 and Figure 6). In conclusion, the use of POC assays for a proactive strategy does not improve the optimization efficacy of IBD patients treated with ADA.

## Figures and Tables

**Table 1:** Clinical characteristics of the first retrospective IBD cohort. IBD: Inflammatory Bowel Disease; CD: Crohn’s disease; UC: ulcerative colitis.

Type of IBD	20 CD/11 UC
Age (years)	30.5 +/− 8 years
Sex Ratio M/F	1.2
Clinical Remission	20 (12 CD)
Other Anti-TNF Before	12 (9 IFX, 3 Golimumab)
Other Treatments with ADA	7 (5 5ASA, 4 steroids)
Duration of Disease (years)	5.2 +/−3.5
Optimization of ADA (40 mg ew)	22

ADA-adalimumab; TNF-tumor necrosis factor; M: Men; F: Female; ASA- Azathioprine; IFX- infliximab.

**Table 2:** Clinical characteristics of the prospective IBD cohort. IBD: Inflammatory Bowel Disease; CD: Crohn’s disease; UC: ulcerative colitis; IFX: infliximab; TNF: tumor necrosis factor; ADA: adalimumab; M: Men; F: Female.

9	*n*
IBD	37
CD/UC	26/11
Age (Years)	34.5 + /−6.5
Duration of Disease (Years)	6.4 +/−3.5
Sex Ratio M/F	1.2
Previous Treatment	Anti-TNF: 12 IFX, 4 Golimumab Vedolizumab: 3 patients Azathioprine: 8 patients
In Therapeutic Optimization	14
In Therapeutic De-Escalation	2
Clinical Remission	18
ADA at 40 mg every week	6
ADA at 40 mg Every Two Weeks	30
ADA at 40 mg Every Three Weeks	1
Adjuvant Treatment with Imurel	3
Anti-ADA Antibodies Detected	2

**Table 3:** Technical characteristics of the different assays used for the measure of ADA trough levels.

Suppliers	Specificity	Range	Dilution	Method	Time (min)
Ridascreen (R-Biopharm)	Monoclonal	0.2–12.6 (μg/mL)	1/100	ELISA	100 min
Lisa-Tracker (Theradiag)	Polyclonal	0.3–16 (μg/mL)	1/200	ELISA	150 min
Lisa-Tracker (Theradiag)	Monoclonal	0.3–C20 (μg/mL)	1/200	ELISA	150 min
Ridaquick (R-Biopharm)	Monoclonal	1–C25 (μg/mL)	1/50	Lateral flow assay	30 min
Quantum Blue (Buhlmann)	Monoclonal	1.3–C35 (μg/mL)	1/20	Lateral flow assay	30 min

**Table 4:** Median and interquartile ranges (IQR, μg/mL) between assays. POC-Point of Care.

	ELISA Theradiag (Polyclonal)	ELISA Theradiag (Monoclonal)	ELISA R-Biopharm	POC Buhlmann	POC R-Biopharm
Number of values	33	33	33	33	33
Minimum	0.3	0.3	0.2	1.3	1.0
25th Percentile	0.3	0.3	0.2	1.3	1.0
Median	2.8	2.2	2.7	2.3	2.3
75th Percentile	7.2	8.5	9.7	11.5	13.9
IQR	4.5	4.7	4.8	6.4	7.9
